# Assessment of Public Health Events through International Health Regulations, United States, 2007–2011

**DOI:** 10.3201/eid1807.120231

**Published:** 2012-07

**Authors:** Katrin S. Kohl, Ray R. Arthur, Ralph O’Connor, Jose Fernandez

**Affiliations:** Centers for Disease Control and Prevention, Atlanta, Georgia, USA (K.S. Kohl, R.R. Arthur, R. O’Connor);; and Department of Health and Human Services, Washington, DC, USA (J. Fernandez)

**Keywords:** public health emergencies of international concern, public health events, international health regulations, disease reporting, United States, IHR

## Abstract

People and goods travel rapidly around the world, and so do infectious organisms. Sometimes a disease has already become widespread before it is detected and reported, which makes control efforts much more difficult. In response to this threat, the World Health Assembly enacted International Health Regulations that require participating countries to report public health events of international concern to the World Health Organization within 72 hours of detection. These health regulations went into effect in 2007 for all WHO Member States including the United States. By December 2011, 24 events reported by the United States were posted on a secure WHO web site, 12 of which were associated with influenza. Others reported were salmonellosis outbreaks, botulism, E. coli infections, Guillain-Barré syndrome, contaminated heparin, Lassa fever, an oil spill, and typhoid fever. International Health Regulations have improved global connectivity through rapid information exchange and increased awareness of threatening situations.

## Medscape CME Activity

Medscape, LLC is pleased to provide online continuing medical education (CME) for this journal article, allowing clinicians the opportunity to earn CME credit.

This activity has been planned and implemented in accordance with the Essential Areas and policies of the Accreditation Council for Continuing Medical Education through the joint sponsorship of Medscape, LLC and Emerging Infectious Diseases. Medscape, LLC is accredited by the ACCME to provide continuing medical education for physicians.

Medscape, LLC designates this Journal-based CME activity for a maximum of 1 *AMA PRA Category 1 Credit(s)*^TM^. Physicians should claim only the credit commensurate with the extent of their participation in the activity.

All other clinicians completing this activity will be issued a certificate of participation. To participate in this journal CME activity: (1) review the learning objectives and author disclosures; (2) study the education content; (3) take the post-test with a 70% minimum passing score and complete the evaluation at www.medscape.org/journal/eid; (4) view/print certificate.

**Release date: June 14, 2012; Expiration date: June 14, 2013**

## Learning Objectives

Upon completion of this activity, participants will be able to:

- Describe overall potential public health emergencies of international concern (PHEIC) from states parties posted by WHO on a secure Web portal between July 2007 and December 2011
- Describe potential PHEIC from the United States posted by WHO on a secure Web portal between July 2007 and December 2011
- Describe potential benefits of having the IHR framework for notification in place, as well as strategies for reporting and sharing information with the WHO

## CME Editor

**Thomas J. Gryczan, MS,** Technical Writer/Editor, *Emerging Infectious Diseases. Disclosure: Thomas J. Gryczan, MS, has disclosed no relevant financial relationships*.

## CME Author

**Laurie Barclay, MD,** freelance writer and reviewer, Medscape, LLC. *Disclosure: Laurie Barclay, MD, has disclosed no relevant financial relationships.*

## Authors

*Disclosures:* ***Katrin S. Kohl, MD, PhD, MPH; Ralph O’Connor, PhD;*** *and* ***Jose Fernandez, PhD,*** *have disclosed no relevant financial relationships.* ***Ray R. Arthur, PhD,*** *has disclosed the following relevant financial relationships: owns stock in Vivus Inc.*
