# Supplementary material for: Assessment of Public Health Events through International Health Regulations, United States, 2007–2011
Source: Emerg Infect Dis. 2012 Jul;18(7):1047–53. doi: 10.3201/eid1807.120231 (PMC3376817; doi:10.3201/eid1807.120231)
Supplement: Related texts

# Review article: Assessment of Public Health Events through International Health Regulations, United States, 2007–2011

**DOI:** 10.3201/eid1807.120231

**Authors:** Katrin S. Kohl, Ray R. Arthur, Ralph O’Connor, Jose Fernandez

**Affiliations:** Centers for Disease Control and Prevention, Atlanta, Georgia, USA (K.S. Kohl, R.R. Arthur, R. O’Connor);; and Department of Health and Human Services, Washington, DC, USA (J. Fernandez)

## Abstract

Under the current International Health Regulations, 194 states parties are obligated to report potential public health emergencies of international concern to the World Health Organization (WHO) within 72 hours of becoming aware of an event. During July 2007–December 2011, WHO assessed and posted on a secure web portal 222 events from 105 states parties, including 24 events from the United States. Twelve US events involved human influenza caused by a new virus subtype, including the first report of influenza A(H1N1)pdm09 virus, which constitutes the only public health emergency of international concern determined by the WHO director-general to date. Additional US events involved 5 *Salmonella* spp. outbreaks, botulism, *Escherichia coli* O157:H7 infections, Guillain-Barré syndrome, contaminated heparin, Lassa fever, an oil spill, and typhoid fever. Rapid information exchange among WHO and member states facilitated by the International Health Regulations leads to better situation awareness of emerging threats and enables a more coordinated and transparent global response.

Global air travel makes it possible for most countries to be reached from a country furthest away within a day, and some countries are connected by direct flights to >70 other countries. Just as persons and goods travel rapidly around the world, so too can pathogens. The outbreak of severe acute respiratory syndrome (SARS) in 2003 continues to symbolize the real possibility of rapid international disease spread of an emerging pathogen (*1*). It also raised awareness that global disease threats can go undetected and unreported to the point that control efforts are extremely difficult because major spread has often already occurred.

The experience with SARS led to the call for more transparent and rapid sharing of information on health risks and public health measures between countries and the World Health Organization (WHO) (*2*). In 2005, the World Health Assembly adopted revised International Health Regulations (IHR) with the declared purpose to “prevent, protect against, control and provide a public health response to the international spread of disease in ways that are commensurate with and restricted to public health risks, and which avoid unnecessary interference with traffic and trade” (*3*). The IHR legally bind 194 WHO states parties, including all WHO member states. One of the key principles inspiring the IHR is open, fast, and secure information exchange about disease emergence and response activities. The IHR provide a platform for dialog in form of national focal points (NFPs), which are always-available points of contacts in each IHR state party for all IHR-related information exchange with WHO and other NFPs, and through provision of a secure web portal, the IHR Event Information Site (EIS), which is accessible by all NFPs.

The IHR went into effect in the United States on July 18, 2007, with the explicit reservation that the United States assumes its obligations “in a manner consistent with its fundamental principles of federalism,” an acknowledgment that responsibilities in the United States under these Regulations are shared between the Federal Government and the States. In addition, the United States specifically understands that all countries have an obligation to notify to WHO potential public health emergencies of international concern (PHEICs) “irrespective of origin or source, whether they involve the natural, accidental or deliberate release of biological, chemical or radionuclear materials” (*4*). In this report, we focus primarily on application of IHR assessment and reporting requirements within the United States for rapid sharing of information on potential PHEICs.

## Assessment and Reporting per IHR Obligation

A critical feature of reporting under the current IHR compared with international reporting requirements detailed in the previous version of the IHR in 1969 is that states parties not only report events on the basis of a prescribed list of diseases, but also on the basis of a list of assessment criteria for any event with the potential for international spread, even if the source or cause of the event is unknown. Annex 2 of the IHR provides the decision instrument for assessing and notifying WHO of a potential PHEIC (*3*). A PHEIC is defined by Article 1 of the regulations as an extraordinary event that may “constitute a public health risk to other States through international spread of disease” and “potentially require a coordinated international response” (Article 1, Definitions, IHR). In addition to any disease with a risk for international spread, certain listed diseases must always be assessed, and 4 diseases (human influenza caused by a new virus subtype, wild-type poliomyelitis, smallpox, and SARS) must always be immediately reported to WHO.

The 4 criteria that guide the assessment are the following: 1) is the public health effect of the event serious?; 2) is the event unusual or unexpected?; 3) is there a major risk for international spread?; and 4) is there a major risk for travel or trade restrictions? If 2 of the 4 criteria are met, the event must be reported to WHO (*3*). The IHR document further provides examples to guide states parties in application of these criteria, and WHO has developed a guidance document for the application of the decision instrument (*5*). Once an event is reported to WHO, information is assessed by WHO and the states parties concerned, and further actions to be taken by WHO are determined. These actions include sharing information about the event with the global community by the secure EIS portal, providing technical assistance, and escalating the assessment to the level of the WHO director-general for considering if the event is determined to be a PHEIC.

Rapid information exchange in the context of the IHR is defined as a 48-hour period for states parties to assess an emerging event, and an additional 24 hours to report the event to WHO, if the assessment indicates that the event may constitute a PHEIC. The 48-hour assessment period begins once the national level of government becomes aware of the event. Just as the report of a potential PHEIC to WHO is meant to be the beginning of a constructive dialogue between states parties and WHO, states parties are obligated to respond to any inquiries from WHO about disease events within their borders within 24 hours, even if those events have otherwise not been reported to WHO. For example, in 2008, the WHO IHR Contact Point for the Americas, hosted by the Pan American Health Organization, requested an assessment of a measles outbreak in the United States as a potential threat to the global measles elimination initiative. Although information regarding this outbreak had already been released (*6*) at the time of the request, the Pan American Health Organization used IHR communication channels to receive a formal assessment to better gauge the US response capability and alert other countries with more in-depth information about a potential threat to their measles elimination activities. The United States provided an assessment that indicated that the outbreak did not meet the criteria required for formal notification as a potential PHEIC.

In another situation, the IHR framework enabled the US public health community to better understand the risk to travelers exposed to a rabid animal in a game resort in Kenya in 2011 (*7*). Because the US government first became aware of this event through returning travelers, and was concerned about travelers from other countries who also may have been exposed, we used the IHR reporting structure to successfully engage WHO to assist with global contact-tracing activities. Because decision criteria for potential PHEICs have been accepted by all IHR states parties, these criteria provided an a priori accepted basis for our rapid and transparent joint assessment with the Kenyan Ministry of Health. Although no formal notification of a potential PHEIC was made to WHO, use of the IHR framework enabled all parties involved to better understand and respond to the exposure risk.

## Assessment of US Public Health Events as Potential PHEICs

During July 2007–December 2011, WHO posted 222 events from 105 member states assessed by WHO on the IHR EIS, including 24 events from the United States (Figure). Half of the events from the United States involved human influenza caused by a new virus subtype (12 events), followed by *Salmonella* spp. outbreaks (5 events) (*8**–**12*) and 1 event each for botulism (*13*), contaminated heparin (*14*), *Escherichia coli* O157:H7 (*15*), an oil spill (*16*), Lassa fever (*17*), Guillain-Barré syndrome (*18*), and typhoid fever (*19*) (Table).

**Figure:** Twenty-four public health events in the United States assessed by the World Health Organization and posted on the International Health Regulations information site, July 2007–December 2011. There was 1 event of botulism and 1 event of *Salmonella* sp. infection in 2007; 1 event related to heparin and 1 event of *Salmonella* sp. infection in 2008; 5 events of influenza, 1 event of *Escherichia coli* infection, and 1 event of *Salmonella* sp. infection in 2009; 3 events of influenza, 1 event of Lassa fever, 1 event related to an oil spill, 1 event of *Salmonella* sp. infection, and 1 event of typhoid fever in 2010; and 4 events of influenza, 1 event of Guillain-Barré syndrome, and 1 event of *Salmonella* sp. infection in 2011.

**Table:** Noninfluenza public health events in the United States, July 2007–December 2011, assessed by WHO as potential public health emergencies of international concern and posted on the IHR secure web portal*

Event by year of assessment (reference)	Assessment determination by WHO per IHR criteria†				Description of event at time of assessment
	1	2	3	4	
2007					
Botulism (*13*)	X	X	X	X	Four cases associated with a canned food product were identified in 2 states for the first time in 40 y in the United States. The company exported food items to >8 countries.
*Salmonella enterica* serovar Wandsworth infection (*10*)	X	X		X	Fifty-seven cases were identified in 18 states in the United States. *Salmonella* spp. can cause serious illness in specific risk groups. Implicated products were sold in the United States and Canada. Other national health authorities are requesting additional information about the outbreak.
2008					
Heparin (*14*)	X		X	X	Contaminated heparin products identified in >2 countries were associated with life-threatening clinical events. The United States Food and Drug Administration enacted an Import Alert for the products.
*S. enterica* serovar Saintpaul infection (*11*)	X	X	X	X	Several hundred cases with the same genetic fingerprint have been identified in 40 states, the District of Columbia, and Canada; illness onset was during April–July 2008. This organism was a previously rare cause of salmonellosis in the United States. The implicated food items (raw hot peppers) grown in Mexico were recalled.
2009					
*Escherichia coli* O157:H7 infection (*15*)	X	X	X		A multistate outbreak in 70 persons (25 were hospitalized, 7 showed development of hemolytic uremic syndrome) was associated with eating raw, refrigerated, prepackaged cookie dough that was exported to numerous other countries.
*S. enterica* serovar Typhimurium infection (*12*)	X	X	X	X	Several hundred cases have been reported in 43 states with an onset during September–December 2008. The outbreak was associated with peanut-containing products, an unusual vehicle for this organism. At least 30 countries may have received the products, and a food recall was implemented.
2010					
Lassa fever (*17*)	X		X		Four days after travel on 3 connecting flights involving 3 continents, a patient was hospitalized for sore throat, diarrhea, thrombocytopenia, and given a diagnosis 5 d later. No high-risk contact was identified, but awareness of travel-associated cases is essential.
Oil spill (*16*)	X	X			An accident at an oil drilling rig off the US coast resulted in an ongoing leak from the well. Modeling suggested that high winds might distribute oil over a wider area, which may potentially affect coastal fisheries and other human activity in other countries.
*S. enterica* serovar Montevideo infection (*8*)	X		X		Several hundred cases were identified in 42 states with an onset during July 2009–January 2010. The implicated food item (salami) was exported to 8 countries and was recalled.
Typhoid fever (*19*)	X	X	X		Nine confirmed cases were reported to CDC from 2 states; 7 persons were hospitalized. Consumption of frozen mamey fruit pulp was epidemiologically linked to the outbreak. The pulp was manufactured in Guatemala and shipped throughout the United States and possibly other countries.
2011					
Guillain-Barré syndrome (*18*)	X	X	X		Twenty-three suspected cases were clustered in time and place along the United States–Mexico border, possibly associated with *Campylobacter jejuni.*
*S. enterica* serovar Enteritidis infection (*9*)		X	X	X	Forty-two cases were reported in 6 states linked to consumption of pine nuts imported from Turkey. The product was recalled.

*WHO, World Health Organization; IHR, International Health Regulations; X, yes; CDC, Centers for Disease Control and Prevention. †1, Is the public health effect of the event serious?; 2, Is the event unusual or unexpected?; 3, Is there a major risk for international spread?; 4, Is there a major risk for international travel or trade restrictions?

Such events may involve no human illness, but must demonstrate the potential risk for human disease. For example, one of the considerations for reporting the oil spill along the US Gulf Coast in 2010 was the potential for a change in ocean currents that may have led to the international dispersion of oil with potential harm to human activities, e.g., coastal fishing. Events may involve only 1 case of disease, e.g., several reports by the Centers for Disease Control and Prevention (CDC) of influenza caused by a new virus subtype involved 1 case; some reports included >2 unrelated cases; and other reports included small clusters of influenza cases. Some events may be assessed when only a few cases are identified, e.g., the outbreak of typhoid fever was assessed when 9 cases from 2 states were confirmed. For other events, hundreds of cases were identified by the time of the assessment. For example, the outbreak of *Salmonella* Typhimurium infection was assessed at a time when >500 patients from 43 states had been identified.

Three events serve as examples for assessment and reporting practices in the United States for potential PHEICs and may assist others in their interpretation of the IHR assessment criteria in the decision instrument. In the first example, the first 2 cases of what later became known as influenza A(H1N1)pdm 09 infection were identified at CDC on April 17, 2009, and reported to WHO by the US Department of Health and Human Services as a potential PHEIC the same day (*20*). WHO determined that the event met all 4 assessment criteria because it involved a new subtype of influenza virus, which was likely to be highly transmissible; the first 2 cases clustered in time without an apparent epidemiologic link; 1 of the case-patients had traveled to Mexico within the incubation period; and major media attention was potentially impairing international travel or trade. On April 25, 2009, the WHO director-general determined that the event constituted a PHEIC on the basis of additional information from the United States and Mexico (*21*), and declared a pandemic 7 weeks later (*22*). No other public health event, including other novel influenza strains reported by the United States or any other country, has so far been determined to be a PHEIC.

The second example involves the second most frequently reported pathogen by the United States under the IHR, i.e., different strains of *Salmonella* spp., in this case, *Salmonella enterica* serotype Enteritidis infection (*9*). On October 27, 2011, CDC reported an outbreak of *S.* *enterica* serotype Enteritidis associated with pine nuts from Turkey as a potential PHEIC to WHO. At the time of reporting to WHO, 42 cases of *S.* *enterica* serotype Enteritidis infection with an identical genetic fingerprint and onset dates during August 20–October 8 had been reported to CDC from 6 states. Nineteen (63%) of 30 patients interviewed had consumed these pine nuts, and ill persons had purchased the pine nuts from bulk bins of the same grocery store chain. During the assessment by senior public health scientists, it was determined that the event was unusual in that pine nuts had not been associated with *Salmonella* spp. outbreaks and thus constituted an unusual vehicle of transmission.

In addition, it was determined that a major risk for international spread and potential for trade restrictions were present because the pine nuts were imported from Turkey and similarly exported to Canada. However, the event did not meet the criterion for a serious effect on public health. Given that *Salmonella* spp. are estimated to contribute to 11% of all domestically acquired foodborne illness and >1 million estimated illnesses each year (*23*), this outbreak was not particularly large compared with other *Salmonella* spp. outbreaks. Because 3 of the 4 assessment criteria from the IHR decision instrument were met, WHO was formally notified of the event. A PHEIC was not determined by WHO, but the event was posted as a WHO-assessed public health risk on the IHR EIS. In the United States, the product was recalled from the grocery store chain, and no new cases were identified 44 days after the beginning of the outbreak.

The third example is a joint report by the United States and Mexico for a binational cluster of cases of acute flaccid paralysis (*18*). At the time of reporting, 23 suspected cases of Guillain-Barré syndrome were identified in a localized area along the United States–Mexico border. Several of the case-patients had evidence of infection with the enteric bacterium *Campylobacter jejuni*, which has been associated with Guillain-Barré syndrome. The event was determined to have a potentially serious effect on public health because several hospitalizations had been reported. The event was also determined to be unusual or unexpected because the local incidence of acute flaccid paralysis had doubled, compared to the expected rate for the same time frame and location. The joint assessment stated that the event posed a major risk for international spread because cases had been reported in the border area in Mexico and in the United States. However, because of localized spread, albeit between 2 countries, the event was not deemed to potentially lead to travel or trade restrictions. At the time the event was reported, because it met 3 of the 4 IHR assessment criteria, the definitive diagnosis, Guillain-Barré syndrome, or the underlying cause for the outbreak (later believed to have been caused by diarrheal illness likely linked to contaminated water systems), were not yet known. This report was not determined to be a PHEIC by WHO, but was posted as a WHO-assessed public health risk on the IHR EIS, as were regular updates on the progress of the outbreak investigation.

The ability of the United States to assess a public health event under the assessment criteria of the IHR decision instrument depends on the following: 1) the federal government becomes aware of an event; 2) federal, state, and local subject matter experts investigating the event are familiar with IHR reporting obligations; 3) and functional surveillance systems are in operation. The ability to determine to report an event requires minimum epidemiologic assessment capacities, including a certain level of expert judgment, and close collaboration with involved parties (e.g., local and state health departments, other federal agencies, or foreign governments). In the United States, we reported >10% of all events posted on the IHR EIS as events assessed by WHO by using the criteria for public health risk for international concern since the IHR went into effect.

Overall, events posted on the IHR EIS represent events that occurred in ≈60% of states parties. Taking into account that the implementation of the IHR is a collective learning process, this might reflect the need to define the purpose of the IHR EIS and be explicit about the threshold for assessment and posting. For example, not all notified events from the United States were posted as WHO-assessed events on the IHR portal, but some were used for public health action by WHO; for example, notifications to WHO of international air travelers with extensively drug-resistant tuberculosis resulted in contact-tracing activities in several countries. In other situations, states parties might be less prone to initiate and sustain a dialog with WHO through the IHR communication channels because of their limited capacity to detect unusual health events or restrictive information sharing policies. An example of this reluctance includes incomplete reporting of new cases of poliomyelitis.

This information signals the need for additional resources to implement the IHR globally. WHO is collaborating closely with its member states to meet IHR requirements for core capacities for surveillance, including the capacity to detect events of potential international public health concern and rapidly assess and report these events to WHO. Although the decision instrument allows for user judgment and experience with resultant lack in specificity (*24*), it can serve as an aid toward the goal of rapid and transparent reporting by states parties. By June 2012, states parties were expected to meet the minimum core capacities for surveillance and response, and development of designated air ports, sea ports, and ground crossings, unless they request a 2-year extension from WHO.

## Contribution of IHR to Global Information Exchange

Although states parties are documenting their progress toward implementation of the IHR requirements, the IHR has already fostered transparency and speed of sharing information on emerging health threats globally. Provision of secure web portals for public health events and designation of NFPs enable access to PHEIC assessments of other countries and enhance direct exchange of public health information between countries. For example, in 2011 in the United States, we were notified directly at least once a month by NFPs in other countries about an outbreak or possible exposure to an infectious disease that might merit public health follow-up by US public health officials, e.g., contacting a traveler about possible exposure to an infectious disease.

The IHR serve as a reminder of our obligation to the global community, which may get lost in an outbreak investigation and staging of domestic control efforts, and provide a framework for WHO to coordinate a globally harmonized response. This obligation was put to test during the influenza A(H1N1)pdm09 virus outbreak, just 2 years after the IHR went into effect. Although the weaknesses of some countries in detecting and reporting novel influenza strains came to light, the level of coordination through regular regional consultations by the WHO director-general and secure and rapid information exchange on the IHR web portal on new cases and response strategies (*25*) were unprecedented and a welcome improvement to the less coordinated response during the SARS outbreak in 2003. In the first 6 months of the influenza A(H1N1)pdm09 outbreak alone, 517 event updates were posted on the IHR web portal. Continuing to strengthen the capacity of WHO member states to detect, analyze, and report emerging health threats remains a priority for WHO.

Many countries do not rely solely on rapid information exchange within the IHR framework or on traditional surveillance systems to learn about emerging health threats in their own or other countries. For example, nontraditional surveillance based on the widespread availability of the Internet and advances in informational technology over the past 15 years that have provided access to media reports can be used as a rich and useful source for early warning of disease threats, even in situations in which the disease or the etiologic agent are unknown. Event-based surveillance has become a critical part of the global biosurveillance programs of WHO (*26*), the US government, and other countries. The IHR provide a common framework for disease detection and information sharing, including confirmation of media-based reports, but also for in-depth consultation and coordinated response for global threats.

## Conclusions

Reporting of potential PHEICs under the IHR framework is not complete when simply counting the number of states parties (n = 105) who reported events that were posted on the IHR web portal in the past 5 years. However, having the IHR framework for notification in place enables improved global connectivity through better situational awareness and built-in global consultation provisions for response. Over time, the global public health community will come to a shared understanding of what merits IHR reporting to WHO, and will build the IHR assessment into their routine detection and response activities. Such a standardized approach in a secure information exchange environment will provide some assurance that not only will persons, goods, and pathogens travel rapidly around the world, but so will information regarding risks to global public health.

## References

[1] Peiris JS, Yuen KY, Osterhaus AD, Stöhr K. The severe acute respiratory syndrome. N Engl J Med. 2003;349:2431–41. 10.1056/NEJMra03249814681510

[2] Cooke FJ, Shapiro DS. Global outbreak of severe acute respiratory syndrome (SARS). Int J Infect Dis. 2003;7:80–5. 10.1016/S1201-9712(03)90001-412839707PMC7128623

[3] World Health Organization. International Health Regulations 2005, 2nd ed. Geneva: The Organization; 2008 [cited 2012 Apr 9]. http://whqlibdoc.who.int/publications/2008/9789241580410_eng.pdf

[4] World Health Organization. Note from the Permanent Mission of the United States of America to the United Nations Office and Other International Organizations accepting the IHRs. 2010 [cited 2012 Apr 19]. http://www.who.int/ihr/usa.pdf

[5] World Health Organization. WHO guidance for the use of Annex 2 of the International Health Regulations. 2010 [cited 2012 Apr 19]. http://www.who.int/ihr/revised_annex2_guidance.pdf

[6] Centers for Disease Control and Prevention. Measles—United States, January 1–April 25, 2008. MMWR Morb Mortal Wkly Rep. 2008;57:494–8.18463608

[7] Obonyo M, Arvelo W, Kadivane S, Orundu M, Lankau E, Munyua P, Exposure to a rabid zebra among tourists and staff at a safari lodge in Kenya, August 2011 [abstract]. In: International Conference on Emerging Infectious Diseases 2012 poster and oral presentation abstracts. Board no. 66. 2012 [cited 2012 May 15]. http://wwwnc.cdc.gov/eid/pdfs/ICEID2012.pdf

[8] Centers for Disease Control and Prevention. Investigation update: multistate outbreak of human *Salmonella* Montevideo infections; 2010 [cited 2012 Apr 19]. http://www.cdc.gov/salmonella/montevideo

[9] Centers for Disease Control and Prevention. Investigation announcement: multistate outbreak of human *Salmonella* Enteritidis infections linked to Turkish pine nuts. 2011 [cited 2012 Apr 19]. http://www.cdc.gov/salmonella/pinenuts-enteriditis/102611/index.html

[10] Centers for Disease Control and Prevention. *Salmonella* Wandsworth outbreak investigation, June–July 2007. 2007 [cited 2012 Apr 19]. http://www.cdc.gov/salmonella/wandsworth.htm

[11] Barton Behravesh C, Mody RK, Jungk J, Gaul L, Redd JT, Chen S, 2008 outbreak of *Salmonella* Saintpaul infections associated with raw produce. N Engl J Med. 2011;364:918–27. 10.1056/NEJMoa100574121345092

[12] Cavallaro E, Date K, Medus C, Meyer S, Miller B, Kim C, *Salmonella* typhimurium infections associated with peanut products. N Engl J Med. 2011;365:601–10. 10.1056/NEJMoa101120821848461

[13] Centers for Disease Control and Prevention. Botulism associated with canned chili sauce, July–August 2007. 2007 [cited 2012 Apr 19]. http://www.cdc.gov/botulism/botulism.htm

[14] World Health Organization. Contaminant detected in heparin material of specified origin in the USA and in Germany; serious adverse events reported; recall measures initiated. 2008 [cited 2012 Apr 19]. http://www.who.int/medicines/publications/drugalerts/Alert_118_Heparin.pdf

[15] Centers for Disease Control and Prevention. Multistate outbreak of *E. coli* O157:H7 infections linked to eating raw refrigerated, prepackaged cookie dough. 2009 [cited 2012 Apr 19]. http://www.cdc.gov/ecoli/2009/0622.html

[16] National Oceanic and Atmospheric Administration. NOAA’s oil spill response. Hurricanes and the oil spill. 2010 [cited 2012 Apr 19]. http://www.nhc.noaa.gov/pdf/hurricanes_oil_factsheet.pdf

[17] Amorosa V, MacNeil A, McConnell R, Patel A, Dillon KE, Hamilton K, Imported Lassa fever, Pennsylvania, USA, 2010. Emerg Infect Dis. 2010;16:1598–600.2087528810.3201/eid1610.100774PMC3294406

[18] Arizona Department of Health Services Director’s Blog. Guillain Barré investigation update. 2011 [cited 2012 Apr 19]. http://directorsblog.health.azdhs.gov/?p=1722

[19] Centers for Disease Control and Prevention. Investigation update: Multistate outbreak of human typhoid fever infections associated with frozen mamey fruit pulp. 2010 [cited 2012 Apr 19]. http://www.cdc.gov/salmonella/typhoidfever/index.html

[20] Centers for Disease Control and Prevention. Swine influenza A (H1N1) infection in two children—southern California, March–April 2009. MMWR Morb Mortal Wkly Rep. 2009;58:400–2.19390508

[21] World Health Organization. Swine influenza. 2009 [cited 2012 Apr 19]. http://www.who.int/mediacentre/news/statements/2009/h1n1_20090425/en/index.html

[22] World Health Organization. World now at the start of 2009 influenza pandemic. 2009 [cited 2012 Apr 19]. http://www.who.int/mediacentre/news/statements/2009/h1n1_pandemic_phase6_20090611/en/index.html

[23] Centers for Disease Control and Prevention. Estimates of foodborne illness. 2011 [ cited 2012 Apr 19]. http://www.cdc.gov/foodborneburden/2011-foodborne-estimates.html

[24] Haustein T, Hollmeyer H, Hardiman M, Harbarth S, Pittet D. Should this event be notified to the World Health Organization? Reliability of the international health regulations notification assessment process. Bull World Health Organ. 2011;89:296–303. 10.2471/BLT.10.08315421479094PMC3066523

[25] World Health Organization. Implementation of the International Health Regulations (2005). Report of the review committee on the functioning of the International Health Regulations (2005) in relation to pandemic (H1N1) 2009. 2011 [cited 2012 Apr 19]. http://apps.who.int/gb/ebwha/pdf_files/WHA64/A64_10-en.pdf.

[26] Heymann DL, Rodier GR. WHO Operational Support Team to the Global Outbreak Alert and Response Network. Hot spots in a wired world: WHO surveillance of emerging and re-emerging infectious diseases. Lancet Infect Dis. 2001;1:345–53. 10.1016/S1473-3099(01)00148-711871807

# Review article: Assessment of Public Health Events through International Health Regulations, United States, 2007–2011

**DOI:** 10.3201/eid1807.120231

**Authors:** Katrin S. Kohl, Ray R. Arthur, Ralph O’Connor, Jose Fernandez

**Affiliations:** Centers for Disease Control and Prevention, Atlanta, Georgia, USA (K.S. Kohl, R.R. Arthur, R. O’Connor);; and Department of Health and Human Services, Washington, DC, USA (J. Fernandez)

## Medscape CME Quiz

## Earning CME Credit

To obtain credit, you should first read the journal article. After reading the article, you should be able to answer the following, related, multiple-choice questions. To complete the questions (with a minimum 70% passing score) and earn continuing medical education (CME) credit, please go to www.medscape.org/journal/eid. Credit cannot be obtained for tests completed on paper, although you may use the worksheet below to keep a record of your answers. You must be a registered user on Medscape.org. If you are not registered on Medscape.org, please click on the New Users: Free Registration link on the left hand side of the website to register. Only one answer is correct for each question. Once you successfully answer all post-test questions you will be able to view and/or print your certificate. For questions regarding the content of this activity, contact the accredited provider, CME@medscape.net. For technical assistance, contact CME@webmd.net. American Medical Association’s Physician’s Recognition Award (AMA PRA) credits are accepted in the US as evidence of participation in CME activities. For further information on this award, please refer to http://www.ama-assn.org/ama/pub/category/2922.html. The AMA has determined that physicians not licensed in the US who participate in this CME activity are eligible for *AMA PRA Category 1 Credits*™. Through agreements that the AMA has made with agencies in some countries, AMA PRA credit may be acceptable as evidence of participation in CME activities. If you are not licensed in the US, please complete the questions online, print the certificate and present it to your national medical association for review.

## Article Title: Assessment of Public Health Events through International Health Regulations, United States, 2007–2011

## CME Questions

**1. Based on the report by Dr. Kohl and colleagues, which of the following statements about International Health Regulations (IHR) and overall potential public health emergencies of international concern (PHEIC) from states parties posted by the World Health Organization (WHO) on a secure Web portal between July 2007 and December 2011 is most likely correct?**

A. Nearly all states parties posted at least 1 PHEIC

B. states parties are obligated to notify WHO of PHEIC within 72 hours of becoming aware of an event

C. Less than 1% of posted PHEIC were from the United States

D. states parties are only required to report natural release of biological materials

**2. Based on the report by Dr. Kohl and colleagues, which of the following statements about events reported by the United States and posted by WHO on a secure Web portal between July 2007 and December 2011 is most likely correct?**

A. 5% of US events involved human influenza caused by a new subtype

B. One of the US PHEIC involved anthrax

C. All of the US PHEIC involved infectious diseases

D. The only PHEIC determined by the WHO Director-General to date was the first report of the 2009 pandemic virus

**3. You are a public health official consulting to the United States about the benefits of and procedures for complying with the 2005 IHR. Based on the report by Dr. Kohl and colleagues, which of the following statements would most likely appear in your report?**

A. The United States is unlikely to be notified by another country’s National Focal Point regarding potential public health threats

B. Notifications from other countries are unlikely to affect US public health decisions

C. The IHR facilitates rapid information exchange among WHO and its Member States, improving situational awareness of emerging threats and coordinated and transparent global response

D. Current strategies for recognizing events to be reported to WHO are well developed and highly standardized

## Activity Evaluation

**Table:** 

1. The activity supported the learning objectives.				
Strongly Disagree				Strongly Agree
1	2	3	4	5
2. The material was organized clearly for learning to occur.				
Strongly Disagree				Strongly Agree
1	2	3	4	5
3. The content learned from this activity will impact my practice.				
Strongly Disagree				Strongly Agree
1	2	3	4	5
4. The activity was presented objectively and free of commercial bias.				
Strongly Disagree				Strongly Agree
1	2	3	4	5
